# Tertiary Lymphoid Structures and Chemokine Landscape in Virus-Positive and Virus-Negative Merkel Cell Carcinoma

**DOI:** 10.3389/fonc.2022.811586

**Published:** 2022-02-10

**Authors:** Motoki Nakamura, Tetsuya Magara, Shinji Kano, Akihiro Matsubara, Hiroshi Kato, Akimichi Morita

**Affiliations:** Department of Geriatric and Environmental Dermatology, Nagoya City University Graduate School of Medical Sciences, Nagoya, Japan

**Keywords:** biomarker, Merkel cell carcinoma (MCC), tertiary lymphoid structure (TLS), tumor micreoenvironment (TME), chemokine, cohort study

## Abstract

Tertiary lymphoid structures (TLSs) are used as biomarkers in many cancers for predicting the prognosis and assessing the response to immunotherapy. In Merkel cell carcinoma (MCC), TLSs have only been examined in MCPyV-positive cases. Here, we examined the prognostic value of the presence or absence of TLSs in 61 patients with MCC, including MCPyV-positive and MCPyV-negative cases. TLS-positive samples had a significantly better prognosis than TLS-negative samples. MCPyV-positive samples had a good prognosis with or without TLSs, and MCPyV-negative/TLS-positive samples had a similarly good prognosis as MCPyV-positive samples. Only MCPyV-negative/TLS-negative samples had a significantly poor prognosis. All cases with spontaneous regression were MCPyV-positive/TLS-positive. We also performed a comprehensive analysis of the chemokines associated with TLS formation using next-generation sequencing (NGS). The RNA sequencing results revealed 5 chemokine genes, *CCL5*, *CCR2*, *CCR7*, *CXCL9*, and *CXCL13*, with significantly high expression in TLS-positive samples compared with TLS-negative samples in both MCPyV-positive and MCPyV-negative samples. Only 2 chemokine genes, *CXCL10* and *CX3CR1*, had significantly different expression levels in the presence or absence of MCPyV infection in TLS-negative samples. Patients with high CXCL13 or CCL5 expression have a significantly better prognosis than those with low expression. In conclusion, the presence of TLSs can be a potential prognostic marker even in cohorts that include MCPyV-negative cases. Chemokine profiles may help us understand the tumor microenvironment in patients with MCPyV-positive or MCPyV-negative MCC and may be a useful prognostic marker in their own right.

## Introduction

Merkel cell carcinoma (MCC) is a rare malignant skin cancer with potentially high immune activity (1).MCC is treated with immune checkpoint inhibitors (ICIs), but the response rate is only about 30% (2), and many patients exhibit no benefit. Useful biomarkers with practical application are waiting to be discovered. The presence or absence of Merkel cell polyoma virus (MCPyV) infection is reported to be closely related to the tumor mutation burden and amount of neoantigens (3, 4). MCPyV-negative, i.e., ultraviolet-induced, MCCs have a higher tumor mutation burden and more neoantigens than MCPyV-positive MCC. Ultraviolet-induced MCCs, however, are not more responsive to ICIs than virus-induced MCCs (5). The presence of tertiary lymphoid structures (TLSs) in the tumor tissue of many cancers is considered to indicate a better prognosis and a good response to ICIs (6, 7). TLSs are ectopic lymphoid tissues found in inflamed, infected, or tumor tissues. TLSs in solid tumors often activate anti-tumor immunity and contribute to the formation of a favorable immune microenvironment against the tumor (8). A previous study reported that the presence of TLSs correlates with a good prognosis in MCC, but the 21 cases examined in that study were all MCPyV-positive cases (9). Here we examined the correlation between the presence or absence of TLSs and prognosis in 61 MCC cases, including both MCPyV-positive and MCPyV-negative cases. In addition, we performed a comprehensive analysis of immunologic factors, including chemokines, associated with TLS formation using next-generation sequencing (NGS). The involvement of TLSs and chemokines in the cancer microenvironment was investigated in patients with MCPyV-positive or MCPyV-negative MCC.

## Materials and Methods

### Cohort Profile

To examine the relationship between TLSs and prognosis in both MCPyV-positive and MCPyV-negative MCC, we examined 71 samples from 61 Japanese patients with MCC diagnosed histologically on the basis of biopsy or surgical resection samples obtained in 9 facilities (see **Supplementary Table 1**). Among the 71 samples, 61 were primary lesions and 10 were metastatic skin lesions. Metastatic lymph nodes and specimens from other organs were excluded. The patients were predominantly female (63.9%) with a mean age of 77.3 years. Primary tumor sites were the head and neck (67.6%), followed by the limbs (27.9%) and trunk (1.6%). Spontaneous regression occurred after biopsy in 5 cases (8.2%). Primary lesions of other cases were surgically removed, treated with radiation therapy, or both. Chemotherapy, e.g., combined treatment with carboplatin and etoposide, was administered in a few cases with distant metastasis. Two cases were treated with the ICI avelumab. Patient characteristics and treatments are summarized in **Table 1**. This cohort mostly overlaps with the cohort in our previous reports (10, 11), and the 41 cases for which RNA sequencing was performed are the same.

**Table 1:** Patient characteristics.

Characteristics		Value
cases		61
samples		71
Age(range)		77.30 (40-98)
Sex		
	Male	22 (36.1%)
	Female	39 (63.9%)
Race		
	Asian(Japanese)	61 (100%)
MCPyV		Cases (n=61)
	Positive	38 (62.3%)
	Negative	23 (37.7%)
Primary Site		Cases (n=61)
	Head&Neck	43 (67.6%)
	Trunk	1 (1.6%)
	Limbs	17 (27.9%)
Lesion		Samples (n=71)
	Primary	60 (84.5%)
	Skin Meta	11 (15.5%)
Stage at collection		Samples (n=71)
	I	24 (33.8%)
	II	23 (32.4%)
	III	16 (22.5%)
	IV	8 (11.3%)
Treatment		Cases (n=61)
	Surgery	15 (24.6%)
	RT	3 (4.9%)
	Surgery+RT	30 (49.2%)
	Surgery+Chemo	2 (3.3%)
	Surgery+RT+Chemo	4 (6.6%)
	Surgery+RT+ICI	2 (3.3%)
	Spontaneous Regression	5 (8.2%)

RT, radiation therapy; Chemo, chemotherapy; ICI, immune checkpoint inhibitor.

### Immunohistochemistry

The presence of MCPyV infection was determined by immunostaining formalin-fixed paraffin-embedded tissue samples obtained by biopsy or surgical resection using the large T-antigen antibody (CM2B4, Santa Cruz Biotechnology, Dallas, TX, USA). For visualization of tumor-infiltrating cells and measurement of PD-L1 expression, indirect immunofluorescence staining was performed using primary antibodies: anti-PD-1 antibody (ab137132, Abcam, Cambridge, UK), anti-PD-L1 antibody (ab205921, Abcam), anti-CD3 antibody (ab17143, Abcam), anti-CD8 antibody (ab17147, Abcam), anti-CD20 antibody (ab78237, Abcam), and anti-CD21 antibody (ab75985, Abcam). Alexa Fluor 488, Alexa Fluor 546, Alexa Fluor 594, and Alexa Fluor 647 (Invitrogen, Waltham, MA, USA) were used as secondary antibodies. The nuclei were stained with 4’,6-diamidino-2-phenylindole (Vector Laboratories, Burlingame, CA, USA). Fluorescence was observed and captured using a fluorescence microscope BZ-X800 (Keyence, Osaka, Japan). TLSs were typically identified as clusters of CD20-positive cells surrounded by CD3-positive cells. Including immature TLSs, in which only a few CD3-positive cells surround a CD20-positive cell cluster, if a lesion had at least one TLS, it was counted as TLS-positive, as previously described (12). The fluorescence intensities of PD-L1 were calculated from 10 randomly selected fields using ImageJ Software (NIH, Bethesda, MD, USA) as previously described (11). After evaluating entire specimens, CD8-positive cells and PD-1-positive cells were counted in several locations having a high density of infiltrating cells, and the mean value was calculated.

### RNA Extraction and Sequencing

RNA extraction and sequencing were performed for 41 randomly selected samples as previously described (11). Tumor tissue was carefully dissected from 3 to 5 undyed formalin-fixed paraffin-embedded tissue sections (4-µm thick) using a scalpel blade and deparaffinized in 640 µl deparaffinization solution (Qiagen, Hilden, Germany). Total RNA was extracted using an AllPrep DNA/RNA FFPE Kit (Qiagen) according to the supplier’s instructions. The RNA integrity number and DV_200_ values were measured using a Bioanalyzer (Agilent Technologies, Santa Clara, CA, USA) to evaluate the quality of the extracted RNA. RNA samples confirmed to be of sufficient quality were reverse-transcribed to cDNA using a SuperScript VILO cDNA Synthesis Kit (Thermo Fisher Scientific, Waltham, MA, USA) after assessing the density using a Qubit 4 Fluorometer (Thermo Fisher Scientific). cDNA samples were amplified and applied to the NGS using a PTC-100 thermal cycler (MJ Research, Watertown, MA, USA) and Ampliseq for the Illumina Immune Response Panel (Illumina, San Diego, CA, USA). After quantifying the library using a Bioanalyzer, NGS analysis was performed using the MiniSeq System (Illumina). Data were uploaded and analyzed on the cloud-based software application BaseSpace Sequence Hub (Illumina). All data were uploaded to the National Center for Biotechnology Information Gene Expression Omnibus database (GSE154938).

### Data Analysis

NGS data were analyzed on the cloud-based software BaseSpace Sequence Hub (Illumina) using the RNA Amplicon application. A clustered heatmap of all samples was generated using the online tool iDEP.91 (http://bioinformatics.sdstate.edu/idep/). Disease-specific survival was calculated as the time that elapsed from sample collection to death from MCC and analyzed using the Kaplan-Meier method. Statistical analyses were performed using Graph Pad Prism 9 (Graph Pad Software, San Diego, CA, USA) and Pharmaco Analyst Software (Humanlife, Tokyo, Japan). Probability values of less than 0.05 were considered statistically significant.

## Results

### Virus-Negative MCC Without TLSs Has a Poor Prognosis

**Figure 1:**
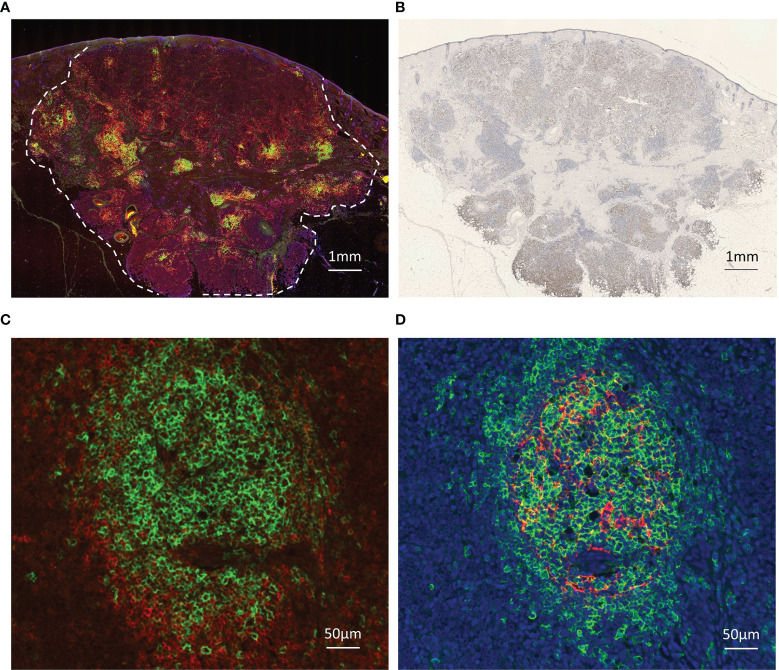
Images of immunohistochemical and immunofluorescent analysis. **(A)** TLSs in Merkel cell carcinoma. Triple immunofluorescence staining for CD20 (green), CD3 (red), and DAPI (blue). Broken line indicates tumor border. Scale bar, 1 mm. **(B)** Immunohistochemical staining for MCPyV large T antigen (CM2B4, brown). Scale bar, 1 mm. TLSs were observed in the stroma inside the tumor, but not in the surrounding area. **(C)** Representative high magnification image of the mature TLS. CD20 (green), CD3 (red), Scale bar, 100 µm. A cluster of CD20-positive cells is surrounded by CD3-positive cells. **(D)** Representative high magnification image of the same mature TLS. CD20 (green), CD21 (red), DAPI (blue). Scale bar, 100 µm. CD21-positive follicular dendritic cells (FDCs) were observed within a cluster of CD20-positive cells.

**Figure 2:**
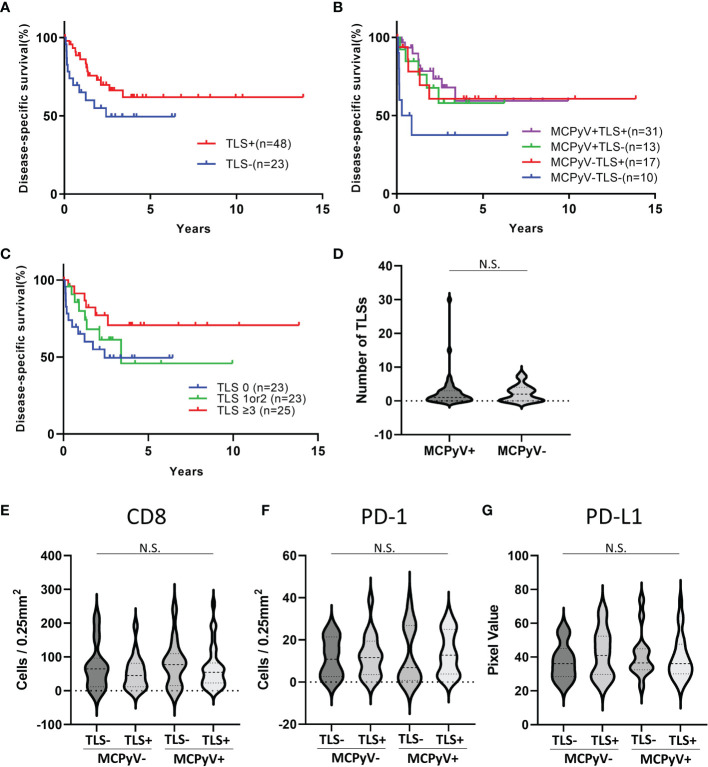
Statistical analyses of immunohistochemical and immunofluorescent analysis. **(A)** Kaplan-Meier curves for the samples with or without TLSs. Gehan-Breslow-Wilcoxon test, p=0.0468. **(B)** Kaplan-Meier curves for the samples with or without TLSs and MCPyV infection. Logrank test for trend, p=0.0497. **(C)** Kaplan-Meier curves for the samples with 0 TLS, 1 or 2 TLSs, and 3 or more TLSs. Logrank test for trend, p=0.0663. **(D)** Violin plots of the number of TLSs with or without MCPyV. There was no significant difference (student T test). **(E–G)** Violin plots of infiltrating lymphocytes and PD-L1 expression. There was no significant difference (one-way ANOVA). N.S., not significant.

The positive rate of MCPyV infection was 62.3% in our Japanese cohort. 48 samples were TLS-positive and 23 samples were TLS-negative. A weak magnification image of a typical tumor is shown in **Figures 1A, B** shows the MCPyV large T antigen staining of the same tumor. This tumor was MCPyV positive. Like this tumor, in many samples, TLSs were observed in the stroma inside the tumor, but not in the surrounding area. Most of the TLSs were immature; mature TLSs with CD3-positive cells circumferentially surrounding a cluster of CD20-positive cells were observed only in a few samples (**Figure 1C**). Only in mature TLSs, CD21-positive follicular dendritic cells (FDCs) were observed within a cluster of CD20-positive cells (**Figure 1D**). TLS-positive samples had a significantly better prognosis than TLS-negative samples (Gehan-Breslow-Wilcoxon test, p=0.0468; **Figure 2A**). When further divided by the presence of MCPyV infection, MCPyV-positive samples had a good prognosis with or without TLSs, and the MCPyV-negative/TLS-positive samples had a similarly good prognosis as MCPyV-positive samples. Only MCPyV-negative/TLS-negative samples had a significantly poor prognosis (Logrank test for trend, p=0.0497; **Figure 2B**). Notably, all samples that showed spontaneous regression were MCPyV-positive/TLS-positive. There was no significant difference between samples with 1 or 2 TLSs and TLS-negative samples (Gehan-Breslow-Wilcoxon test, p=0.249), but samples with more than 3 TLSs showed a significantly better prognosis than TLS-negative samples (Gehan-Breslow-Wilcoxon test, p=0.0413, **Figure 2C**). There was no correlation between the presence of TLS and MCPyV infection (p=0.60, Fisher’s exact test, 2-tailed), and there was no correlation between the number of TLSs and MCPyV infection (student T test, **Figure 2D**).

The number6 of infiltrating CD8-positive cells and PD-1-positive cells did not differ significantly between samples with and without TLS or MCPyV infection (one-way ANOVA, **Figures 2E, F**). The intensity of PD-L1 expression in the tumors also did not differ significantly between samples with and without TLS or MCPyV infection (one-way ANOVA, **Figure 2G**).

### Comprehensive RNA Sequencing

**Figure 3:**
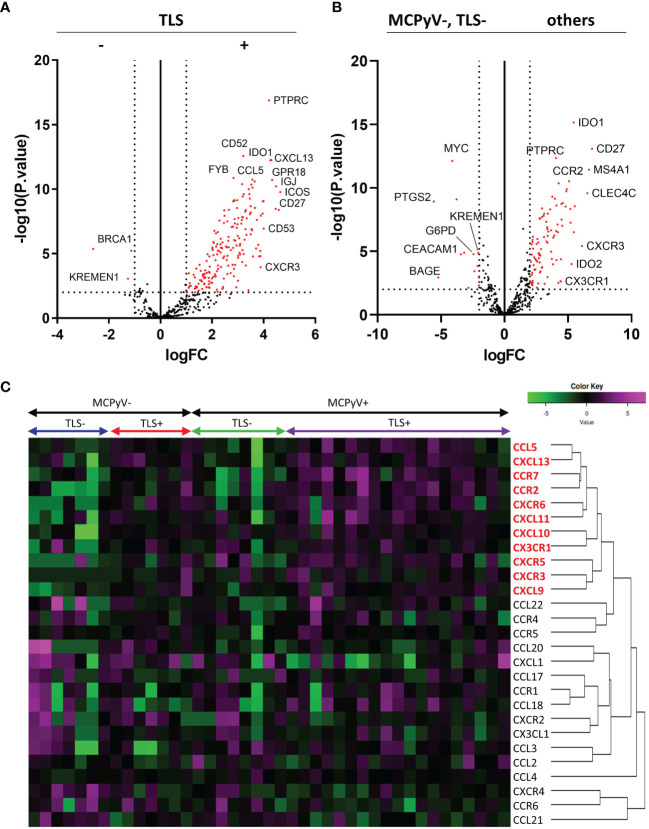
Results of the RNA sequencing. **(A)** Volcano plots comparing TLS-positive and TLS-negative cases. Vertical dotted lines indicate logFC=+/-1. Horizontal dotted line indicates -log10 (p.value)=2. **(B)** Volcano plots comparing MCPyV-negative, TLS=negative cases and others. **(C)** RNA expression heatmap of 27 chemokine and chemokine receptor genes. The chemokines shown in red are elevated in the TLS-positive samples.

Comprehensive RNA sequencing of 395 immune-related genes revealed high expression of some genes, such as *PTPRC*, *IDO1*, and *CD52*, and some chemokine genes, including *CXCL13, CCL5*, and *CXCR3*, in the TLS-positive samples (**Figure 3A**). Comparison of MCPyV-negative/TLS-negative samples with others revealed that several genes, such as *MYC, PTGS2, KREMEN1, G6PD, DEACAM1*, and *BAGE*, were highly expressed in the MCPyV-negative/TLS-negative samples. In other samples with a good prognosis, including TLS-positive samples and TLS-negative/MCPyV-positive samples, we observed upregulated expression of *IDO1, IDO2*, and *CD27*, and some chemokine genes, including *CCR2*, *CXCR3*, and *CX3CR1*, as well as *PTPRC* (encoding CD45) and *MS4A1* (encoding CD20), which encode cell surface proteins of tumor-infiltrating lymphocytes (**Figure 3B**).

### Chemokine Landscape in TLS Formation in Virus-Positive or Virus-Negative MCC

**Figure 4:**
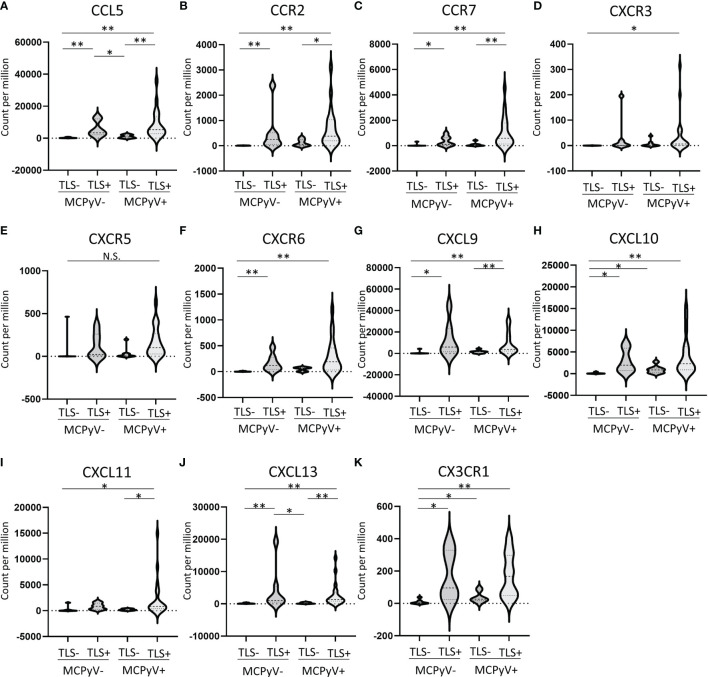
**(A–K)** Violin plots of 11 chemokine genes that were highly expressed in TLS-positive samples. *p ≤ 0.05, **p ≤ 0.01, N.S., not significant; Steel-Dwass test.

A total of 27 chemokine and chemokine receptor genes included in the Illumina Immune Response Panel were analyzed. Hierarchical cluster analysis revealed that 11 chemokine genes (5 ligands and 6 receptors), including *CXCL13* and *CCR7*, were highly expressed in TLS-positive samples (**Figure 3C**). The details of each as violin plots and results of statistical analyses are shown in **Figure 4**. The details of 16 other chemokine genes are shown in **Supplementary Figure 1**. In the TLS-positive samples, 5 chemokine genes, *CCL5*, *CCR2*, *CCR7*, *CXCL9*, and *CXCL13*, were significantly upregulated compared with TLS-negative samples in both MCPyV-positive and MCPyV-negative samples. Only two of them, CXCL13 and CCL5, were barely affected by the presence or absence of MCPyV, but only by the presence or absence of TLSs. On the other hand, 2 chemokine genes, *CXCL10* and *CX3CR1*, had significantly different levels of expression in the presence or absence of MCPyV infection in TLS-negative samples.

### Chemokines May Be a Useful Prognostic Marker in Their Own Right

**Figure 5:**
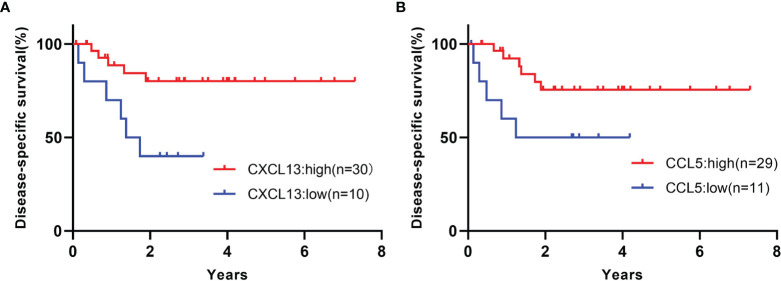
**(A)** Kaplan-Meier curves for the samples of high or low *CXCL13* expression. Gehan-Breslow-Wilcoxon test, p=0.0116. **(B)** Kaplan-Meier curves for the samples of high or low *CCL5* expression. Gehan-Breslow-Wilcoxon test, p=0.0202. These analyses were performed on 40 samples, excluding one case of unknown prognosis, from the 41 samples that underwent RNA extraction.

The expression values of *CXCL13* and *CCL5* were separated by the presence or absence of TLSs, and the receiver operating characteristic (ROC) curves were drawn and the cutoff values were set at 142 count per million (CPM) for *CXCL13* and 547.5 CPM for *CCL5*. CXCL13-high samples showed significantly better prognosis than CXCL13-low samples (Gehan-Breslow-Wilcoxon test, p=0.0116, **Figure 5A**). CCL5-high samples also showed significantly better prognosis than CCL5-low samples (Gehan-Breslow-Wilcoxon test, p=0.0202, **Figure 5B**). These analyses were performed on 40 samples, excluding one case of unknown prognosis, from the 41 samples that underwent RNA extraction.

## Discussion

Our findings indicate that the presence of TLSs is a potential prognostic marker, even for MCPyV-negative cases. To our knowledge, this study is the first to analyze the relationship between the presence of TLSs, MCPyV infection, and prognosis in MCC. A previous study indicated that MCPyV-positive MCC has a better prognosis than MCPyV-negative MCC (13). In the present study, MCPyV-positive patients had a good prognosis with or without TLSs. On the other hand, MCPyV-negative cases had a similar prognosis to MCPyV-positive cases if TLSs were present, but a significantly worse prognosis if TLSs were not present.

TLSs are small lymphoid follicle-like structures that appear around various types of inflammation and cancer, apart from the primary lymph nodes of the thymus and bone marrow, and secondary lymph nodes such as lymph nodes, tonsils, and Peyer’s plate. It serves as a front base for antigen presentation and lymphocyte activation, reflecting an active immune response in the local microenvironment. When TLSs form inside or around tumors, they activate anti-tumor immunity, and make immunotherapy more effective (6). According to a report by Hiraoka et al. in pancreatic cancer, TLS formed within the tumor has a more favorable prognosis than those formed around it (14). The presence or absence of TLS in MCC is important not only as a prognostic marker, but also as a predictive marker of response to ICIs. The relationship between TLSs and the presence or absence of MCPyV infection, which is a unique and immunologically interesting factor in MCC, has not been studied before. MCPyV-positive MCC shows active tumor immunity and comparable responsiveness to immunotherapy, despite having lower TMB and fewer neoantigens than UV-induced MCCs (3–5). The present study also revealed that the prognosis of MCPyV-positive MCC is not affected by the presence or absence of TLSs. It deserves further investigation and a prospective study should be conducted to investigate the correlation with the actual effect of ICIs.

The present study also suggested associations between many immunological factors and TLSs or MCPyV. For example, based on the RNA sequencing results, *G6PD* was significantly upregulated in MCPyV-negative/TLS-negative samples. Glucose-6-phosphate dehydrogenase (G6PD) is a factor that we previously reported as a promising prognostic and immune activity biomarker in MCC (15). Thus, in the present study using the same cohort, *G6PD* was highly expressed, indicating a poor prognosis as well as low immune activity in this group. On the other hand, expression of *IDO1*, *IDO2*, and *CD27* is significantly upregulated in TLS-positive samples. Indoleamine 2,3-dioxygenase (IDO) is an immune checkpoint that induces regulatory T cells and suppresses tumor immunity (16, 17), and its high expression correlates with a poor prognosis in several cancers (18, 19). This paradoxical upregulation of IDO in the group with a good prognosis may be a response to increased tumor immune activity as well as PD-L1 expression in MCC (10). CD27 is a member of the tumor necrosis factor receptor superfamily that plays an important role in T cell activation. Its agonistic antibody in combination with ICI therapy is expected to be effective against MCC (20).

A variety of chemokines are involved in the formation of TLSs (21, 22). In particular, CXCL13 is expressed on PD-1–positive lymphocytes and FDCs present in B-cell follicles plays a key role in the formation of TLSs by inducing the migration of B cells having CXCR5 as a receptor (23–25). CXCR5 is also expressed on T cells and CXCL13 mediates T cell recruitment to TLSs (26). Our results revealed that *CXCL13* was expressed with high specificity in TLS-positive samples. Although there was no significant difference in the expression of *CXCR5*, clustering analysis showed a similar trend. The significant difference observed only in *CXCL13* expression and not in *CXCR5* may be due to the immaturity of many of the TLSs observed in MCCs. On the other hand, in the CCL21/CCL19-CCR7 axis, which is considered to be as important for the formation of TLSs as the CXCL13-CXCR5 axis, high expression of CCR7 was only observed in TLS-positive cases, and no increase in expression of CCL21 was observed (CCL19 was not measured because it was not included in the panel used). These findings support previous reports that CCL21 expression is restricted to lymphatic vessels and does not contribute to TLS formation in the skin (27, 28).

Contrary to the sharply enhanced chemokine expression in TLS-positive samples, the numbers of infiltrating CD8-positive cells and PD-1-positive cells, and PD-L1 expression in tumors did not differ significantly between patients with and without TLSs. In addition, as mentioned above, the presence or absence of TLSs was not associated with the prognosis in MCPyV-positive cases. This finding suggests that MCPyV-positive MCC has a mechanism to activate tumor immunity that does not require TLSs. The chemokines having significantly upregulated expression in the presence or absence of MCPyV may provide a clue. In this study, *CX3CR1* and *CXCL10* were significantly upregulated in MCPyV-positive/TLS-negative samples compared with MCPyV-negative/TLS-negative samples. CX3CR1 and its ligand CX3CL1 play both a role in activating anti-tumor immunity and in promoting tumor formation and progression (29). High expression of CX3CL1-CX3CR1 enhances the recruitment of CD8+ cytotoxic T cells, natural killer cells, and dendritic cells, and results in a better prognosis (30, 31). On the other hand, the CX3CL1-CX3CR1 axis induces angiogenesis and assists in cancer growth (32). In skin cancer (basal cell carcinoma, squamous cell carcinoma), it is expressed in tumor-associated macrophages and is deeply involved in carcinogenesis (33). CXCL10 binds to CXCR3 (it is also upregulated in TLS-positive samples), which is abundantly expressed on cytotoxic T cells and natural killer cells (34). CXCL10 activates anti-tumor immunity by inducing interferon gamma (35), and inhibits angiogenesis and prevents tumor growth (36). Tumor cell lines with high expression of CXCL10 exhibit suppressed growth (37). Although the roles of these chemokines in MCPyV-positive MCC remain unclear, they may contribute to improve the anti-tumor immune environment and a favorable prognosis even without the formation of TLSs.

In MCPyV-negative MCC, which is ultraviolet-induced and has many genetic mutations and neoantigens, the presence or absence of TLS formation is directly related to patient prognosis. On the other hand, MCPyV-positive MCCs seem to have a different mechanism of tumor immune activation. Elucidation of this mechanism will help us understand tumor immunity in MCC, which is strange compared to other tumors.

## Data Availability Statement

The datasets presented in this study can be found in online repositories. The names of the repository/repositories and accession number(s) can be found below: https://www.ncbi.nlm.nih.gov/, GSE154938.

## Ethics Statement

The studies involving human participants were reviewed and approved by Clinical Research Management Center, Nagoya City University Hospital. Written informed consent for participation was not required for this study in accordance with the national legislation and the institutional requirements.

## Author Contributions

MN contributed to conception and design of the study, performed experiments and statistical analysis, wrote the first draft of the manuscript. TM performed experiments and statistical analysis. SK and AMa performed experiments. HK and AMo contributed to manuscript revision. All authors read and approved the submitted version of the manuscript.

## Funding

This work was supported by a Grant-in-Aid for Scientific Research (C) from the Ministry of Education, Culture, Sports, Science, and Technology, Japan (No.20K08676) and the Japan Agency for Medical Research and Development (AMED) under grant (No. JP20cm0106301h0005, presented to: Hiroyoshi Nishikawa, National Cancer Center).

## Conflict of Interest

MN received honorarium for lecturing at seminars held by companies such as Merck Biopharma Co., Ltd.

The remaining authors declare that the research was conducted in the absence of any commercial or financial relationships that could be construed as a potential conflict of interest.

## Publisher’s Note

All claims expressed in this article are solely those of the authors and do not necessarily represent those of their affiliated organizations, or those of the publisher, the editors and the reviewers. Any product that may be evaluated in this article, or claim that may be made by its manufacturer, is not guaranteed or endorsed by the publisher.

## Supplementary Material

Supplementary Table 1 The 9 facilities where the samples were collected.

Supplementary Figure 1
**(A–P)** Violin plots of 16 other chemokine genes that were not highly expressed in TLS-positive samples. *p ≤ 0.05, **p ≤ 0.01, N.S., not significant; Steel-Dwass test.
