# Supplementary material for: Tertiary Lymphoid Structures and Chemokine Landscape in Virus-Positive and Virus-Negative Merkel Cell Carcinoma
Source: Front Oncol. 2022 Feb 10;12:811586. doi: 10.3389/fonc.2022.811586 (PMC8867579; doi:10.3389/fonc.2022.811586)
Supplement: Supplementary Table 1 — The 9 facilities where the samples were collected. [file Table_1.docx]

Supplementary Table 1

| The 9 facilities where the samples were collected |
| --- |
| Gifu Prefectural General Medical Center |
| Gunma University Hospital |
| Kanazawa University Hospital |
| Nagaoka Red Cross Hospital |
| Nagoya City University Hospital |
| Osaka City University Hospital |
| Saitama Medical University Hospital |
| Saitama Medical University International Medical Center |
| Yokohama City University Hospital |
