# Supplementary figures and images for: Tertiary Lymphoid Structures and Chemokine Landscape in Virus-Positive and Virus-Negative Merkel Cell Carcinoma
Source: Front Oncol. 2022 Feb 10;12:811586. doi: 10.3389/fonc.2022.811586 (PMC8867579; doi:10.3389/fonc.2022.811586)

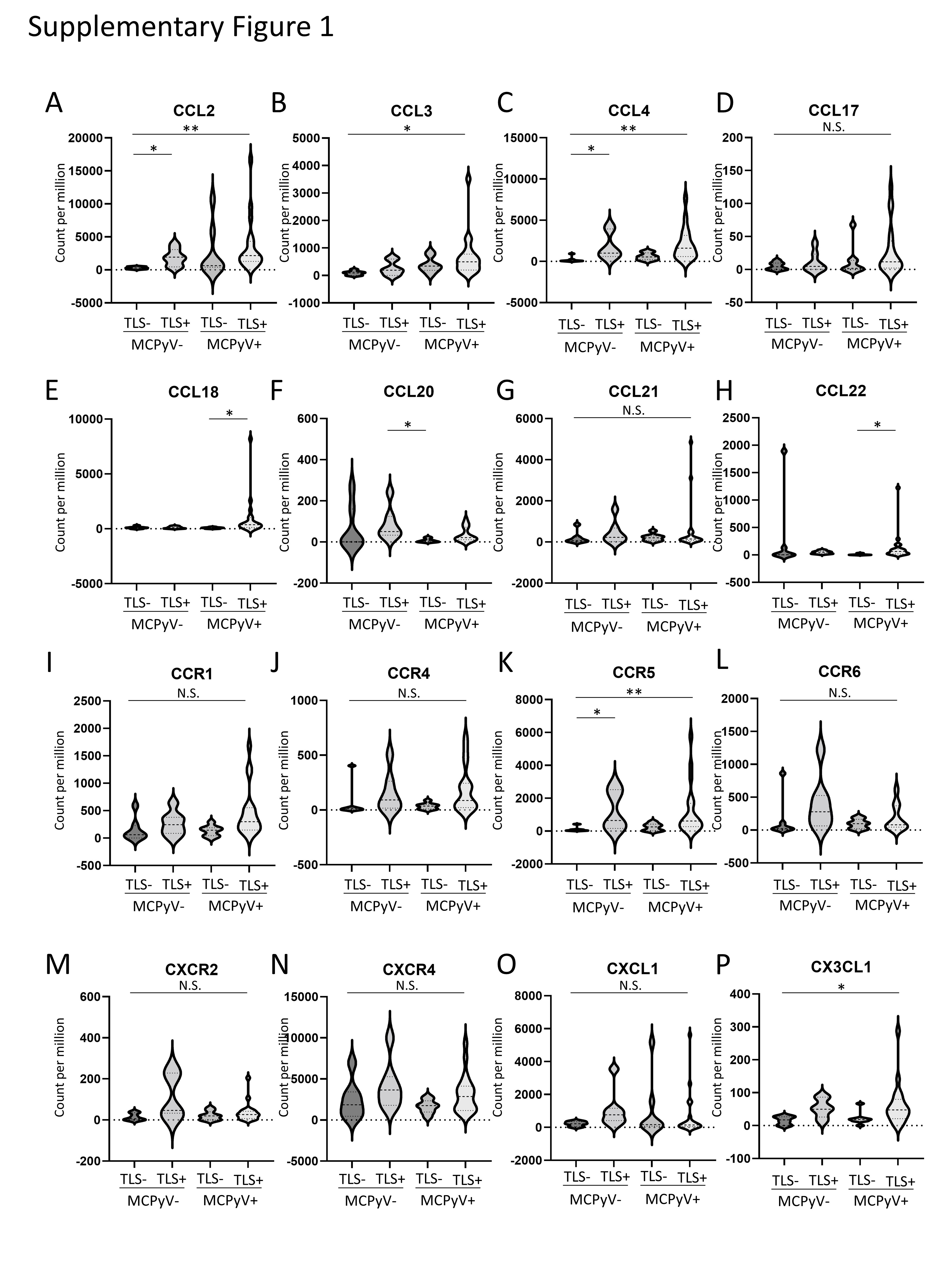

Supplement: Supplementary Figure 1 — (A–P) Violin plots of 16 other chemokine genes that were not highly expressed in TLS-positive samples. *p ≤ 0.05, **p ≤ 0.01, N.S., not significant; Steel-Dwass test. [file Image_1.jpeg]
